# Machine-learning based classification of 2D-IR liquid biopsies enables stratification of melanoma relapse risk†

**DOI:** 10.1039/d5sc01526j

**Published:** 2025-04-07

**Authors:** Kelly Brown, Amy Farmer, Sabina Gurung, Matthew J. Baker, Ruth Board, Neil T. Hunt

**Affiliations:** a Department of Chemistry and York Biomedical Research Institute, University of York UK; b School of Medicine and Dentistry, University of Central Lancashire UK; c Department of Oncology, Lancashire Teaching Hospitals NHS Trust Preston UK neil.hunt@york.ac.uk

## Abstract

Non-linear laser spectroscopy methods such as two-dimensional infrared (2D-IR) produce large, information-rich datasets, while developments in laser technology have brought substantial increases in data collection rates. This combination of data depth and quantity creates the opportunity to unite advanced data science approaches, such as Machine Learning (ML), with 2D-IR to reveal insights that surpass those from established data interpretation methods. To demonstrate this, we show that ML and 2D-IR spectroscopy can classify blood serum samples collected from patients with melanoma according to diagnostically-relevant groupings. Using just 20 μL samples, 2D-IR measures ‘protein amide I fingerprints’, which reflect the protein profile of blood serum. A hyphenated Partial Least Squares-Support Vector Machine (PLS-SVM) model was able to classify 2D-protein fingerprints taken from 40 patients with melanoma according to the presence, absence or later development of metastatic disease. Area under the receiver operating characteristic curve (AUROC) values of 0.75 and 0.86 were obtained when identifying samples from patients who were radiologically cancer free and with metastatic disease respectively. The model was also able to classify (AUROC = 0.80) samples from a third group of patients who were radiologically cancer-free at the point of testing but would go on to develop metastatic disease within five years. This ability to identify post-treatment patients at higher risk of relapse from a spectroscopic measurement of biofluid protein content shows the potential for hybrid 2D-IR-ML analyses and raises the prospect of a new route to an optical blood-based test capable of risk stratification for melanoma patients.

## Abstract 2

Ultrafast 2D-IR spectroscopy of clinical blood serum samples combined with Machine Learning shows the ability to detect risk of melanoma relapse.

## Graphical abstract

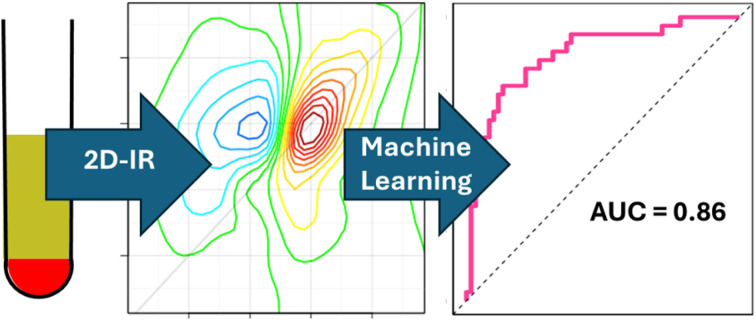

## Introduction

Non-linear spectroscopy methods based on ultrafast lasers, such as two-dimensional infrared (2D-IR) spectroscopy, are capable of measuring large, information-rich datasets from a given molecular sample.^1^ Applications of 2D-IR methods have revealed considerable new insights into molecular structure,^2^ dynamics,^3–5^ intermolecular interactions^6,7^ and reactions.^8–14^ The information density of 2D-IR arises from the ability to spread the vibrational spectrum of a molecule over a second frequency dimension, somewhat akin to 2D-NMR, along with the introduction of a time-resolved axis that reports on ultrafast dynamics. 2D-IR has found considerable applications to proteins where its sensitivity to intramolecular vibrational couplings and energy transfer leads to a protein amide I band shape that is highly susceptible to changes in protein secondary structure and dynamics, including subtle effects such as those resulting from ligand binding.^15–28^ Taken together with the close biological link between a protein's structure and its function, this means that the amide I 2D-IR spectrum of a protein can be considered to be a unique, label-free, fingerprint of its solution-phase structure.

In parallel with progress in 2D-IR interpretation, the last decade has also seen considerable advances in measurement technology, with high pulse repetition-rate lasers and mid-IR pulse shaping meaning that a 2D-IR spectrum now takes just seconds or minutes to acquire.^29–33^

This combination of information density and data abundance makes 2D-IR a promising candidate for combination with data science approaches such as machine learning (ML) to maximise the insight obtained from experimental datasets. The possibility of linking ML with 2D-IR has been assessed using simulated data, showing the potential for models to learn spectral signatures of dynamic proteins.^34,35^ Experimental applications of ML to 2D-IR have also shown the ability to classify spectra of small, purpose-designed sets of chemically distinct samples.^25^

An important barrier for hybrid 2D-IR-ML approaches to cross however is to provide insights from experimental data that could not be achieved with traditional spectroscopic analyses. The ability to approach problems that are intractable by other means would open the door to many new applications in protein analysis ranging from structure interpretation and intermolecular interactions to biomedical analysis.^36^ To explore this, we have linked 2D-IR protein fingerprints and ML to classify blood serum samples collected from patients with melanoma according to their protein profile.

Human serum is a protein-rich fluid, containing around 70 mg mL^−1^ proteins composed mainly of serum albumin (35–50 mg mL^−1^) and the globulins (25–35 mg mL^−1^).^37^ The latter group is comprised of more than 50 individual types of protein, present at concentrations ranging from milligrams to less than micrograms per millilitre. The types and concentrations of proteins present in blood serum samples respond sensitively to metabolic processes^37^ and, of relevance to this study, the protein profile can also be a marker for disease.^38–40^

The range and varying abundance of constituent proteins also mean that measuring the serum protein profile quickly and directly is challenging. Infrared (IR) absorption spectroscopy studies have highlighted changes in protein signatures in samples from cancer patients,^41^ but a combination of a lack of resolution and confounding absorptions from water hinder direct interpretation of protein signals.^42^ Despite this, studies using sample drying or background subtraction methods have shown that IR signatures of blood serum samples can be used to detect cancers and have reported changes in the protein region of the spectrum, but detailed analysis was restricted to bands assigned to non-proteinaceous species.^41–49^ In contrast to IR absorption, 2D-IR not only spreads the protein signature over two spectral dimensions, increasing resolution, but also suppresses the background water absorption^27^ allowing a more direct and detailed measurement of changes in serum protein profiles without sample manipulation or background subtraction.

Here, we apply 2D-IR and ML to the problem of melanoma risk stratification. Melanoma is the fifth most common cancer in the UK, with incidences rising worldwide. A major challenge in treatment planning for melanoma patients is the accurate assessment of the post-operative risk of relapse. Patients at high risk of developing melanoma metastasis (relapse) after surgery can reduce the risk and increase their distant melanoma-free survival through adjuvant treatment.^50–53^ Whilst adjuvant therapies, both immunotherapy and BRAF-targeted treatments, reduce the recurrence risk, more work is required to distinguish patients needing treatment from those cured by surgery alone.^54^ This is important to healthcare providers in terms of reducing treatment burden and the high price of drugs, but vital to patients who could avoid treatment toxicities if adjuvant therapy is not required. Furthermore, melanoma patients at high risk of relapse undergo regular radiological imaging for five years post-surgery, irrespective of adjuvant therapy.^55^ This exposes patients to serial radiation, which increases the risk of cancer. The ability to identify patients with high-risk disease through alternative methods would therefore improve follow up stratification.

The diagnostic process to establish a patient's risk of relapse currently depends simply on the stage of the melanoma. A liquid biopsy, using biofluids to identify at-risk patients would therefore provide a step-change in early detection, leading to lifesaving and prolonging treatment whilst avoiding treatment toxicities in others. Our results show that a hybrid 2D-IR-ML approach is capable of differentiating serum samples according to diagnostically relevant groups. The considerable overlap of the spectra in these groups means that such an outcome would be extremely difficult without the application of ML tools and so highlights the potential of such methods. Although exploratory, our results also suggest that optical tools based on advanced spectroscopies and ML could have a role to play in future diagnostic approaches.

## Experimental

### 2D-IR spectroscopy

The two-dimensional infrared (2D-IR) spectrometer featured two Yb-based amplified lasers (Pharos 20 W and Pharos 10 W, Light Conversion) synchronized by a common oscillator.^56^ Each laser was used to pump an optical parametric amplifier (OPA, Orpheus Mid-IR, Light Conversion) equipped with difference frequency generation to produce independently tuneable sources of pump and probe pulses respectively for 2D-IR spectroscopy.

For the experiments described below, the output of both OPAs was centred at 1650 cm^−1^, resonant with the protein amide I mode. The OPAs produced usable bandwidths of >200 cm^−1^ with energies of 2.5 and 1.5 μJ per pulse, respectively, at a pulse repetition rate of 50 kHz.

2D-IR data collection was *via* a 2DQuick spectrometer (Phasetech) employing the pump–probe beam geometry and a mid-IR pulse shaper to generate and control the time delay (*τ*) between the pair of “pump” pulses.^57,58^ Signal detection was *via* 64-element HgCdTe array detector using the ZZZZ (parallel) polarization geometry, which maximises signal intensity. Each sample was measured at waiting time (*T*_w_) values of 250 fs and 5 ps, yielding both the protein signal (*T*_w_ = 250 fs) and a small thermal signal from H_2_O (*T*_w_ = 5 ps) that was used for signal pre-processing and standardisation *via* previously published methods.^27,59,60^ For a given value of *T*_w_, *τ* was scanned in steps of 24 fs to a maximum delay time of 3 ps, applying a rotating frame frequency of 1208 cm^−1^. Each 2D-IR plot represents the average of 500 spectra, repeated 3 times.

### Patient samples

Samples were sourced from the study Spectroscopic Diagnosis of Melanoma, REC reference number 15/LO/1312, approved by London-Brent. Samples were collected from patients with a confirmed diagnosis of melanoma. Blood samples were anonymised and serum extracted *via* centrifugation. After extraction, serum was stored at −80 °C. Non-identifiable clinical and demographic data were obtained in-line with the study protocol.

The serum samples were representative of three patient groups: the *control* group, where after surgery the patient did not present with a subsequent cancer diagnosis. The *metastatic* group where the presence of metastatic disease was already confirmed at the time the blood sample was obtained, and the *developed metastasis* group, where patients were radiologically cancer free following surgery but went on to develop metastatic disease within the five-year follow-up period. The sample cohort analysed consisted of 40 individual patients; 8 *control*, 21 *metastatic* and 11 *developed metastasis*. A breakdown of the relevant patient metadata for each class is given in Table S1.†

### Sample measurement

For each sample, 20 μL of defrosted serum was placed between two CaF_2_ windows, without the inclusion of a spacer. The pathlength of the cell was adjusted so that the absorbance of the *δ*_H–O–H_ + *υ*_libr_ combination band of water located at ∼2130 cm^−1^ was equal to ∼0.1, corresponding to a sample thickness of ∼2.75 μm.^27^

Each patient sample was measured in triplicate, generating three spectra per patient. To account for potential variations in instrument performance with time, *control* group spectra were collected during each measurement set, resulting in the measurement of 16 *control* samples, with each of the 8 individual *control* patients' serum measured twice. Overall, this resulted in the collection of 144 spectra. 48 spectra in the *control* group, 63 in the *metastatic* group and 33 in the *developed metastasis* group.

### Data pre-processing

After 2D-IR measurements, spectral pre-processing was performed using the previously published workflow,^59^ utilising custom R scripts. In brief, this involves using the thermal water response at *T*_w_ = 5 ps to perform baseline correction and signal normalisation.^59,60^ This normalisation procedure corrects for pathlength variations and instrument variability between measurements. Savitsky–Golay smoothing was also applied. All spectra were processed in unison and with the same pre-processing parameters to ensure no spectral processing variations would be introduced.

### Algorithm training and cross validation

Machine learning models were developed utilising the *Caret* and *pROC* packages in R to identify the unique spectral fingerprint associated with samples from each of the three classes: *control*, *metastatic* and *developed metastasis*. This model was then used to classify spectra from a blind set of patient samples. The predictive classification model used a nested cross-validation (CV) framework that incorporated partial least squares (PLS) for dimensionality reduction and a support vector machine (SVM) for classification, a general schematic of the ML process is shown in Fig. S1.† A 3-fold outer CV was implemented using unique patient identifiers, with training and test splits generated to maintain class balance across the folds. For each outer fold, the training data was further partitioned in the inner loop for hyperparameter tuning, which employed a 3-fold CV within the training data. At all stages, the dataset was rigorously stratified based on sample ID to ensure that no replicate spectra from the same patient appeared in both testing and training sets.

PLS was employed to address the high dimensionality of the spectral dataset by projecting the scaled and mean centred spectral data onto a lower-dimensional latent variable (LV) space while maximising covariance with the class labels. PLS was applied independently to each training and test split to extract 15 LVs representing the most informative spectral features. During execution of the nested CV, the overlap within the feature space between the training and testing PLS LV scores was assessed by comparing the distribution of PLS scores for the training and test sets in each outer fold. This evaluation confirmed that the test and training sets within each outer-fold produced scores of similar magnitudes, validating the suitability of this approach within the nested CV PLS-SVM model (Fig. S2†). The extracted LV scores were then used as input features for training the SVM models with a radial basis function (RBF) kernel. Hyperparameters for the cost parameter (*C*) and sigma were optimised using a grid search strategy with area under the one-*vs.*-all receiver operating characteristic curve (AUROC) of the validation sets used to guide parameter selection.

For each outer fold, the final SVM model was trained on the full training set with the optimal hyperparameters obtained from the inner loop. Model performance was assessed using the independent test set using Cohen's kappa, sensitivity, specificity and AUROC parameters. Probabilistic predictions were recorded to facilitate *post hoc* analysis and visualisation of class separations. Variable importance in projection (VIP) scores were calculated for each PLS LV to assess their contribution to the model. VIP scores were computed by weighting each component's contribution to the explained variance of the PLS model. The use of the nested CV approach allowed for unbiased estimates of generalisation performance but also ensured model tuning and evaluation were conducted on strictly independent datasets. By employing a stratified, hierarchical framework, we mitigate the risk of overfitting, especially given the imbalanced dataset.

## Results and discussion

### 2D-IR spectra

**Fig. 1:**
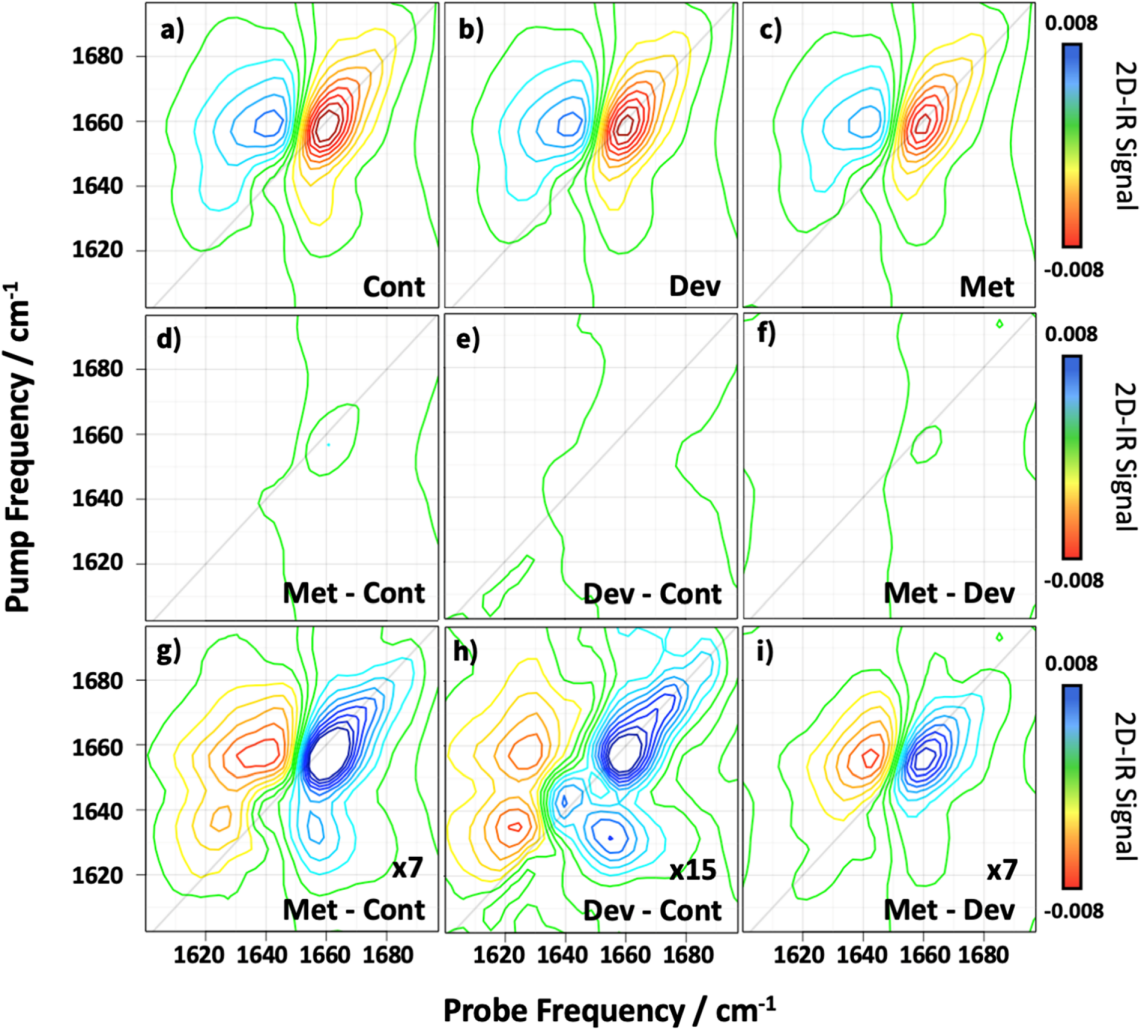
Average 2D-IR spectra obtained with *T*_w_ = 250 fs for each of the different patient classification groups (a) control (Cont, 48 spectra), (b) developed metastasis (Dev, 33 spectra) and (c) metastatic (Met, 63 spectra). Difference spectra determined for subtraction of the averaged spectra of the three classes from each other are shown in panels (d) metastatic – control, (e) developed metastasis – control and (f) metastatic – developed metastasis. (g) to (i) show the difference spectra from (d)–(f) expanded by the multiplication factor shown.

The 2D-IR spectra of the collected serum samples in the amide I region (Fig. 1(a–c)) show a band shape that is consistent with previous studies using commercial, pooled serum.^25–27,60^ A clear negative band (red) is present on the spectrum diagonal near 1660 cm^−1^ which is assigned largely to the amide I *v* = 0–1 transition of the predominant α-helix-rich human serum albumin protein. The positive (blue) peak due to the accompanying, anharmonically-shifted, *v* = 1–2 transition is located near a probe frequency of 1640 cm^−1^.^27^ The 2D peak shapes are asymmetric, extending in a teardrop shape towards pump frequencies of 1630–1640 cm^−1^ as a result of contributions from the globulin protein family, which contain a greater fraction of β-sheet structures compared to serum albumin.^27^ Although descriptive assignments of the features are possible, it is important to note that these spectra represent an additive profile arising from the amide I bands of all proteins present in the sample, scaled by their respective concentrations, and so are more effectively thought of as a fingerprint of the protein profile for each patient sample. As such, variations in amide I peak shape or intensity between patient groups could reveal differences in protein profile (types, concentrations, structures, post-translational modifications, aggregation), potentially arising from disease states or progression. Realistic assessments of the sensitivity of 2D-IR to proteins in H_2_O-rich media suggest that the 2D-IR protein fingerprint should be sensitive to fluctuations in the contributions of around 10–12 of the most abundant proteins by concentration.^44,59^

The spectra in Fig. 1(a)–(c) show averaged results encompassing all of the spectra measured from patients in each of the three groups (*control* (a), *developed metastasis* (b) and *metastatic* (c)). The spectra are broadly similar, as would be expected given the general similarities of human protein profiles, though some small differences are apparent in the amplitude and shape of the amide I bands in Fig. 1(a–c). Difference spectra (Fig. 1(d–f)) produced *via* subtraction of the spectra in Fig. 1(a–c) from one another show that the spectral changes between classes only appear clearly following magnification (Fig. 1(g–i)), revealing the subtle distinctions between the patient groups. This broad consistency between samples confirms the effectiveness of the data pre-processing strategy. It is encouraging to note that the changes displayed in Fig. 1(g–i) focus not only on the α-helix region of the spectrum, near 1660 cm^−1^, but also in the β-sheet region near 1630–1640 cm^−1^. This firstly suggests that the changes between samples are localised on the protein portion of the response, rather than spectral noise for example. Secondly, it suggests that there may be changes in both albumin and globulin content that can be used to differentiate spectra of the three sample groups.

Although we can extract these subtle changes through averaging of all spectra from a given group and careful spectral subtraction, visual classification on a per-sample basis would be challenging and unreliable, a fact that would be further complicated by patient-to-patient and sample-to-sample variation as protein levels respond to many everyday factors. The overlapping spectral features, combined with variations in peak intensity and shape, create an intricate pattern that does not lend itself to straightforward interpretation, as demonstrated by the application of PCA or PLS analyses (Fig. S3†). However, ML models offer a solution *via* the ability to identify patterns within complex datasets. By training models on a range of spectral data, the ability to detect subtle spectral variations can be developed, potentially enhancing classification accuracy. Our aim was thus to exploit ML methods to leverage the nuanced spectral information, improving diagnostic reliability that could ultimately reveal markers of disease progression or risk from the serum protein profile.

### Machine learning development

A number of ML methods were tested to address the challenges of classifying 2D-IR spectra of serum samples according to their diagnostic groups. 2D-IR spectra of the amide I region (1600–1700 cm^−1^) contain some 2624 pixels, not all of which contain information that will be useful for classification. As a result, hyphenated ML approaches exploiting partial least squares (PLS) to perform dimensionality reduction of the data were employed. The initial attempt used PLS-Discriminant Analysis (PLS-DA). This supervised technique combines PLS dimensionality reduction with classification by identifying latent variables (LV) that maximise group separation while minimising noise from irrelevant spectral features.^25,61^ PLS-DA is particularly well suited toward high dimensionality data such, as 2D-IR spectra where co-linearity among variables can complicate analysis. While PLS-DA showed some ability to separate the three sample groups, its overall classification performance was limited, though sufficient clustering in some LVs suggested that more advanced machine learning strategies could improve the classification accuracy (Fig. S4†).

Subsequently, more powerful classification approaches such as *k*-Nearest Centroid (kNC), Random Forest (RF) and Support Vector Machines (SVM) were evaluated due to their proven efficacy for high-dimensional dataset classifications.^62–67^ All three hyphenated models (with PLS) were implemented using the nested CV approach described in the experimental section. The performance of each model was assessed using the standard evaluation metrics of AUROC, sensitivity and specificity. Each of these metrics provide unique insights into the model's classification performance. AUROC evaluates the model's ability to distinguish between classes, with values closer to unity indicating better discriminating power. Sensitivity assesses the ability to identify true positives correctly, which is crucial for detecting subtle spectral differences, while specificity evaluates the ability to identify true negatives correctly, reflecting the model's robustness in minimising false positives.

The performance of the three models is summarised in Table 1, where classification performance of the *control*, *developed metastasis* and *metastatic* groups is shown. The kNC model demonstrated moderate improvements in sensitivity and AUROC compared to PLS-DA for the *developed metastasis* and *metastatic* groups, although its performance for the *control* group remained limited. The RF model further improved AUROC and specificity, particularly for the *metastatic* group, but its sensitivity for the *control* and *developed metastasis* groups remained below commonly accepted performance standards.

**Table 1:** Summary of model performance metrics of all PLS-X models assessed. All metrics reported are the average obtained across 3 outer folds of the nested CV

Model	Parameter	Sample group		
		*Control*	*Developed metastasis*	*Metastatic*
*k*-Nearest centroid (kNC)	**AUROC**	**0.53**	**0.66**	**0.73**
	Sensitivity	0.53	0.56	0.93
	Specificity	0.78	0.96	0.71
Random Forest (RF)	**AUROC**	**0.63**	**0.70**	**0.81**
	Sensitivity	0.36	0.50	0.93
	Specificity	0.82	0.90	0.69
Support vector machine (SVM)	**AUROC**	**0.75**	**0.80**	**0.86**
	Sensitivity	0.69	0.72	0.70
	Specificity	0.76	0.89	0.88

The SVM model emerged as the most effective approach, achieving the highest AUROC values across all groups (0.75, 0.80 and 0.86 for *control*, *developed metastasis* and *metastatic*, respectively). PLS-SVM also achieved a balance between sensitivity and specificity, with notable improvements in sensitivity for the *control* and *developed metastasis* groups. These results therefore show that SVM offers the most promising approach for addressing the classification challenges posed by the 2D-IR dataset.

### PLS-SVM model performance

**Fig. 2:**
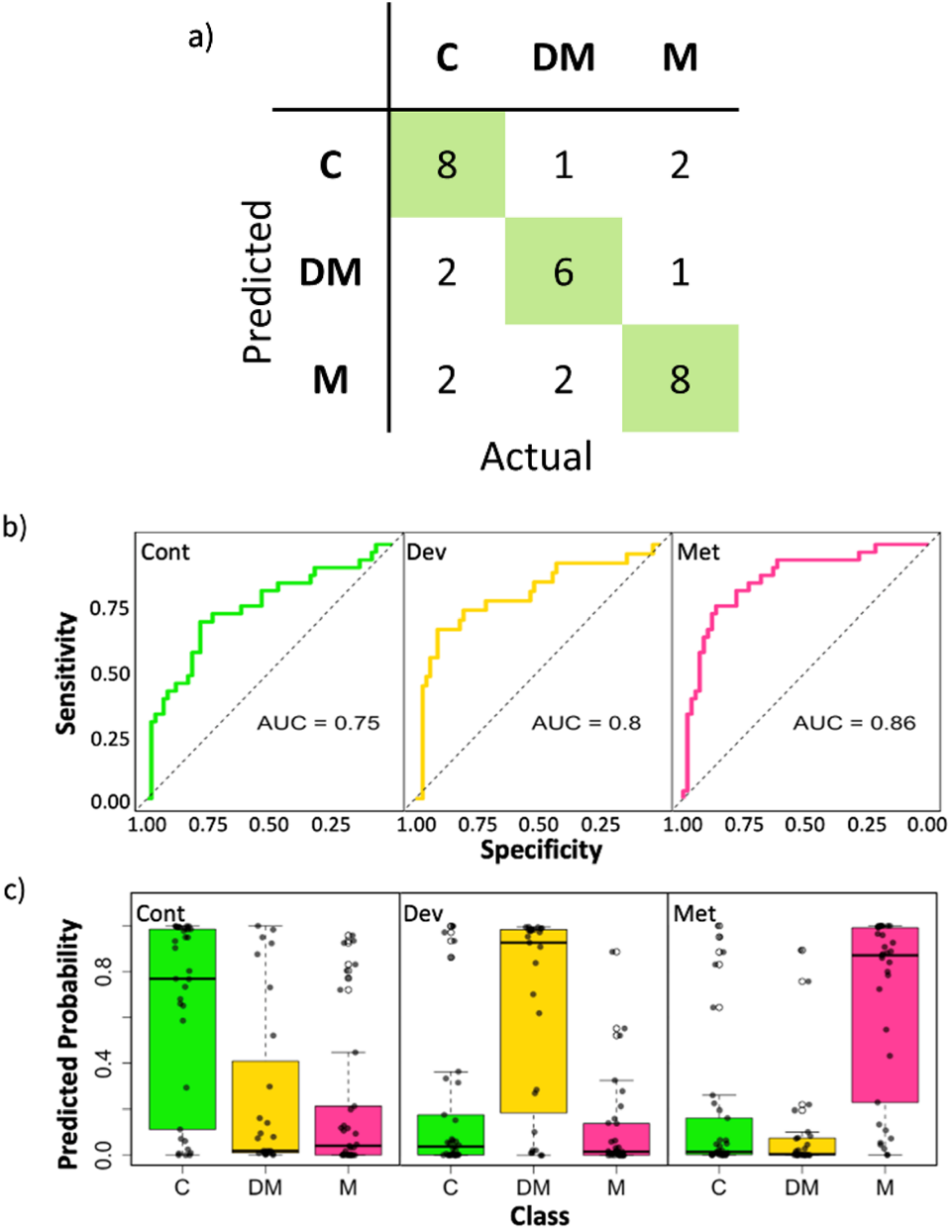
Average performance metric outputs across outer folds for test samples, (a) average confusion matrix, (b) average ROC curves for each class: control (green), developed metastasis (gold) and metastatic (pink) (c) prediction probability box plots across all outer folds for each class, with jitter points added showing each individual probability value and range. Clear separation of the target category from the others show the model's confidence in producing a classification.

To gain a deeper understanding of the classification performance of the PLS-SVM model, the average performance across the three outer folds of the nested CV model is presented in Fig. 2. The individual fold performances are also provided in the ESI (Fig. S5–S7†). The average confusion matrix (Fig. 2(a)) demonstrates that the PLS-SVM model is able to differentiate between samples from the three patient groups. Correct predictions, shown along the matrix diagonal, clearly dominate while misclassifications are relatively evenly distributed across the three groups. The even distribution of misclassifications also indicates that the model does not exhibit a systematic bias toward any particular class. Instead, the errors appear to stem from the natural variability in the dataset rather than model-specific shortcomings. The latter observation is consistent with spectral variations between the groups that are overlapping, as indicated by PCA and PLS analyses. Note that the total number of samples listed in the matrix reflect just the portion of the data set that appears in the outer fold test set.

The ROC curves (Fig. 2(b)) for each sample group further demonstrate the discriminative power of the PLS-SVM model, with AUROC values of 0.75, 0.80, and 0.86 for the *control*, *developed metastasis*, and *metastatic* groups, respectively. These values show that the model effectively separates the classes, particularly for the *metastatic* group, where the highest AUROC value reflects superior classification performance. The shape of the ROC curves for all groups, with an upward trajectory towards the top left corner of the plot, indicates high sensitivity and specificity across the range of classification thresholds. This progression highlights the ability of the model to classify true positives correctly while minimising false positives. The model achieves balanced sensitivity and specificity values across all groups (Table 1), with sensitivity values ranging from 0.69 for the *control* group to 0.72 for the *developed metastasis* group. Specificity values are higher, peaking at 0.89 for the *developed metastasis* group. These results indicate that the model is capable of correctly identifying true positives but also robust in minimising false positives. The Kappa value of 0.523 reflects a more moderate agreement between predicted and actual classifications but is still consistent with reliable performance of the model.

While the PLS-SVM model clearly captures the underlying patterns in the data, the inherent overlap in spectral features would be expected to impose a limitation on classification accuracy for this relatively small experimental dataset. This can be assessed *via* probability box plots (Fig. 2(c)), which provide a quantitative measure of predictive confidence returned by the model for each sample. These plots show that for each of the three groups there is a significant clustering of high probabilities for the correct class, showing that the model maintains strong confidence in its predictive ability. However, the box plots also illustrate the challenge posed by the overlapping spectral features, which results in a relatively wide distribution of probabilities showing lower confidence in some of the predictions. For example, the control group exhibits a broad distribution of predicted probabilities, with significant overlap into the *developed metastasis* and *metastatic* regions. Similarly, the *developed metastasis* group demonstrates a range of probabilities, perhaps reflecting its intermediate nature between the other two groups and so the potential for shared spectral characteristics with the *control* and *metastatic* classes. These overlapping distributions align with the misclassifications observed in the confusion matrix and highlight the presence of some uncertainty in distinguishing between groups but overall, the performance is strong, and uncertainties would be expected to be reduced with the addition of more data to the model.

**Fig. 3:**
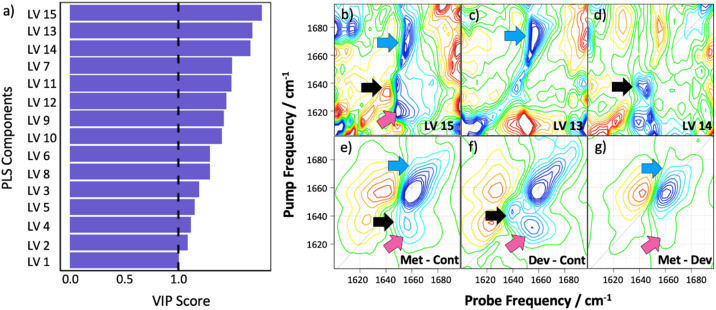
(a) Average variable importance of projection scores across training set of outer folds. VIP scores greater than 1 indicate important latent variables used in making model predictions. Panels (b) to (d) shows the spectral loadings of the three variables considered to be the most important, (b) = LV 15, (c) = LV 13, (d) = LV 14. Panels (e–g) reproduce the difference spectra from Fig. 1(g–i) for comparison. Coloured arrows highlight points of interest as discussed in the text.

It is instructive to consider the regions of the 2D-IR response that the ML model uses to make decisions when classifying samples. Variable Importance in Projection (VIP) scores show the contribution of each PLS LV to the model's classification performance. This not only provides useful spectroscopic insight but can also be used to assess whether classification was based on meaningful, biologically relevant spectral features, rather than random noise and to guard against overfitting. The VIP scores, Fig. 3(a), highlight the importance of the specific PLS components in distinguishing between the *control*, *developed metastasis* and *metastatic* groups. Components with VIP scores greater than unity are considered the most influential, as they capture significant variations in the data and reflect the spectral regions that contribute most to the model. Here we observe that the most important LVs identified are 15, 13 and 14, with LV 15, capturing the most significant variations. The corresponding loading plots (Fig. 3(b–d)) illustrate the specific spectral regions associated with these LVs. The most prominent features in LV 15 and 13 primarily lie in the region around 1660 cm^−1^ with additional contributions from the 1640 cm^−1^ region. LV 14 appears to be dominated by changes in 1625–1640 cm^−1^ region. It is noteworthy that, although no direct correlation between these loadings and the difference spectra discussed above are expected, or necessary for good model performance, many of the areas that arise in LVs 13–15 align with features in the inter-group difference spectra from Fig. 1 (reproduced in Fig. 3(e–g), see coloured arrows). These observations underline that the PLS loadings are identifying regions of the 2D-IR spectrum that correspond to the main parts of the amide I band, as would be expected for a model that is using spectral information for sample classification. In combination with the other parameters that are used to assess model performance this further confirms that the ML approach is leading to an accurate and robust sample classification output.

## Discussion

The use of ML strategies to extract information from 2D-IR spectra and specifically to classify spectra from groups of clinical serum samples is encouraging. While ML approaches have been used for more straightforward classifications of experimental spectra, where clear changes between classes are anticipated,^25^ this represents the first application to a system where it was not clear at the outset that differences would be forthcoming. As such, this provides evidence for the potential of ML approaches to extract information that might not otherwise be recoverable. This finding also adds experimental weight to studies, that have paired ML with simulated 2D-IR data^35^ and should serve to motivate work to enlarge experimental datasets or to explore the use of mixed simulated/experimental strategies.

The fact that our 2D-IR-ML method is able to differentiate spectra obtained from different patient groups based on the serum protein profile is equally encouraging. The method requires only small volumes of blood serum, with measurement times on the order of minutes, while data collection requires no prior sample manipulation to account for the presence of water, all of which suggest that 2D-IR-ML methods have the potential for further development towards applications in biomedical diagnostics and more generally for solution-phase protein analysis.

Considering the results of this study in the more specific context of risk stratification for the treatment of melanoma. A promising approach to detecting melanoma residual disease exploits detection of circulating tumour DNA (ctDNA).^54,68,69^ Presence of ctDNA as a biomarker has been shown to correlate with relapse risk^68^ and clinical trials are ongoing, though quantities of ctDNA in cases of non-metastatic melanoma are small and so hard to detect.^54^ Our results show that variations in the protein profile of the patient's blood serum may offer another, parallel, route to identifying disease states and predicting relapse risk. This correlates with observations relating to other cancers using IR absorption spectroscopy,^41–49^ but the addition of superior spectral resolution means that 2D-IR may offer a useful complementary technology to these tools.

One advantage of the 2D-IR ML approach is the detailed insight that is contained within the regions of the amide I band that were identified with sample classifications. Fig. 3 shows that changes to both the α-helix and β-sheet region were highlighted by the model as being of importance, suggesting that the changes could encompass a range of proteins. It is also noteworthy that some consistency was achieved between ML output and the difference spectra obtained from the average signal from each sample class. This suggests that 2D-IR results may ultimately be able to point towards molecular markers for disease based on changes in the broad protein profile of serum samples. As discussed above, these changes may include variations in the relative concentrations of some of the major proteins, but there are also indications from prior 2D-IR studies of serum that changes in structure, dynamics or ligand binding can all influence the amide I profile.^15^ Equally, the contribution of post-translational modifications to the amide I band are as yet unexplored. In making a link between serum spectroscopy and disease, one has to be aware of potential confounding factors given that serum reports on many bodily processes,^70^ but these results offer a firm basis for follow-up studies. Further experiments using protein libraries to understand some of the potential spectroscopic contributions would be instructive. Similarly, combining 2D-IR results with powerful supporting technologies like proteomics analyses would be of particular value in identifying specific molecular changes that are leading to the 2D-IR-based classifications. Such a multi-platform approach could add vital new information relating to understanding the molecular nature of disease progression.^71^

One clear result of the study is that there is overlap of spectral features between the individual sample groups and that this has proved a challenge for the ML model. This overlap in spectral features was expected given the broad molecular similarity of patient serum samples, the presumed gradual progression of disease states and the shared biochemical markers likely to be present between the groups. For instance, the differences between *control* and *developed metastasis* samples may stem from subtle changes in protein profile that are indicative of early-stage disease. However, since the *developed metastasis* samples come from different patients at varying stages of disease progression, these subtle differences may not be consistently evident across all samples, making it harder to differentiate them from the *control* group. This in essence is the challenge that the 2D-IR-ML approach sought to overcome, so indications that it may be possible are encouraging. The fact that the ML model uses the full 2D-plot also shows that the information density inherent in the 2D-R method will be valuable in doing so.^72,73^ Similarly, patients in the later stages of *developed metastasis* may exhibit spectral profiles that resemble those of *metastatic* disease, further blurring the distinction between these two groups and complicating classification. As has been shown to be the case with ML-based approaches, such problems would benefit considerably from larger studies involving many more samples and serial samples over time.^74,75^ Additionally, the provision of true controls from healthy individuals would provide useful insights. In this respect, the clear differentiation between the three patient groups, all of which have had treatment from melanoma that would be expected to reduce the variation between them is another positive indicator for the potential of combined 2D-IR-ML strategies.

## Conclusions

Here we present a first attempt to apply 2D-IR spectroscopy to the analysis of clinical biofluid samples, specifically targeting the classification of patient serum samples from post-treatment melanoma patients. By leveraging the water suppression protocol of 2D-IR we were able to obtain high quality spectra of the protein amide I region, allowing investigation of whether this response may contain markers for disease progression. By integrating 2D-IR spectroscopy with ML strategies, we have developed a model that could successfully classify patient samples according to three clinically relevant groups: *control*, *developed metastasis* and *metastatic*, establishing proof of principle for the specific application and for future hybrid 2D-IR-ML-based protein analysis strategies.

Despite the nuanced spectral differences observed, manual classification was not tractable due to overlapping spectral features and subtle variations across patient groups. However, advanced ML strategies, particularly the PLS-SVM model, proved capable of good classification performance, achieving AUROC values of 0.75, 0.80, and 0.86 for the *control*, *developed metastasis* and *metastatic* groups, respectively and demonstrating robust discriminative power. Balanced sensitivity and specificity further reinforced the model's reliability in identifying disease states.

These findings highlight the potential of 2D-IR spectroscopy combined with ML to contribute to cancer diagnostics. While the inherent overlap in spectral features imposes some limitations on classification accuracy, the demonstrated ability to differentiate between patient groups at an accepted level underscores the feasibility of this approach for clinical applications. Future work should focus on refining ML strategies, particularly through the expansion of datasets, including the addition of non-symptomatic healthy individuals. These could potentially be enhanced by inclusion of data collected using different polarisation geometries, which could enhance off-diagonal regions of the spectrum, though careful consideration of how to combine the datasets would be required.^76^ Additionally, exploring complementary spectroscopic techniques could enhance classification performance and provide deeper insights into the biological features of disease progression leveraged for classification. Ultimately, this study lays a foundation for the exploration of 2D-IR-ML approaches, offering a promising tool for harnessing the information content of 2D datasets.

## Data availability

Spectral data for this article are available from the research data repository of the University of York at doi: 10.15124/cbcd7bde-7f42-4722-a17d-a1c295d5e444. Data collected from human participants are not available for confidentiality reasons.

## Author contributions

Kelly Brown: investigation, data curation, formal analysis, validation, visualization, writing original draft. Amy Farmer: validation, writing – review and editing. Sabina Gurung: investigation, writing – review and editing. Matthew J. Baker: funding acquisition, methodology, validation, supervision, writing – review and editing. Ruth Board: funding acquisition, methodology, resources, validation, writing – review and editing. Neil T. Hunt: funding acquisition, methodology, validation, supervision, writing – review and editing.

## Conflicts of interest

There are no conflicts to declare.

## Supplementary Material

SC-OLF-D5SC01526J-s001

## References

[cit1] Hamm P. and Zanni M. T., Concepts And Method Of 2D Infrared Spectroscopy, Cambridge University Press, Cambridge, 2011

[cit2] Khalil M., Demirdoven N., Tokmakoff A. (2003). J. Phys. Chem. A.

[cit3] Chung J. K., Thielges M. C., Fayer M. D. (2011). Proc. Natl. Acad. Sci. U. S. A..

[cit4] Ishikawa H., Kwak K., Chung J. K., Kim S., Fayer M. D. (2008). Proc. Natl. Acad. Sci. U. S. A..

[cit5] Ishikawa H., Finkelstein I. J., Kim S., Kwak K., Chung J. K., Wakasugi K., Massari A. M., Fayer M. D. (2007). Proc. Natl. Acad. Sci. U. S. A..

[cit6] Kim Y. S., Hochstrasser R. M. (2005). Proc. Natl. Acad. Sci. U. S. A..

[cit7] Bandaria J. N., Dutta S., Nydegger M. W., Rock W., Kohen A., Cheatum C. M. (2010). Proc. Natl. Acad. Sci. U. S. A..

[cit8] Hamm P. (2021). J. Chem. Phys..

[cit9] Cervetto V., Hamm P., Helbing J. (2008). J. Phys. Chem. B.

[cit10] Kolano C., Helbing J., Kozinski M., Sander W., Hamm P. (2006). Nature.

[cit11] Zhang X. X., Jones K. C., Fitzpatrick A., Peng C. S., Feng C. J., Baiz C. R., Tokmakoff A. (2016). J. Phys. Chem. B.

[cit12] Sanstead P. J., Stevenson P., Tokmakoff A. (2016). J. Am. Chem. Soc..

[cit13] Jones K. C., Peng C. S., Tokmakoff A. (2013). Proc. Natl. Acad. Sci. U. S. A..

[cit14] Chung H. S., Ganim Z., Jones K. C., Tokmakoff A. (2007). Proc. Natl. Acad. Sci. U. S. A..

[cit15] Hunt N. T. (2024). Accounts Chem. Res..

[cit16] Hunt N. T. (2009). Chem. Soc. Rev..

[cit17] Ganim Z., Chung H. S., Smith A. W., Deflores L. P., Jones K. C., Tokmakoff A. (2008). Accounts Chem. Res..

[cit18] Fields C. R., Dicke S. S., Petti M. K., Zanni M. T., Lomont J. P. (2020). J. Phys. Chem. Lett..

[cit19] Alperstein A. M., Ostrander J. S., Zhang T. Q. O., Zanni M. T. (2019). Proc. Natl. Acad. Sci. U. S. A..

[cit20] Buchanan L. E., Dunkelberger E. B., Tran H. Q., Cheng P. N., Chiu C. C., Cao P., Raleigh D. P., de Pablo J. J., Nowick J. S., Zanni M. T. (2013). Proc. Natl. Acad. Sci. U. S. A..

[cit21] Grechko M., Zanni M. T. (2012). J. Chem. Phys..

[cit22] Manor J., Mukherjee P., Lin Y.-S., Leonov H., Skinner J. L., Zanni M. T., Arkin I. T. (2009). Structure.

[cit23] Hamm P., Lim M., Hochstrasser R. M. (1998). J. Phys. Chem. B.

[cit24] Shaw D. J., Waters L. C., Strong S. L., Schulze M., Greetham G. M., Towrie M., Parker A. W., Prosser C. E., Henry A. J., Lawson A. D. G., Carr M. D., Taylor R. J., Hunt N. T., Muskett F. W. (2023). Chem. Sci..

[cit25] Rutherford S. H., Hutchison C. D. M., Greetham G. M., Parker A. W., Nordon A., Baker M. J., Hunt N. T. (2023). Anal. Chem..

[cit26] Rutherford S. H., Greetham G. M., Towrie M., Parker A. W., Kharratian S., Krauss T. F., Nordon A., Baker M. J., Hunt N. T. (2022). Analyst.

[cit27] Hume S., Hithell G., Greetham G. M., Donaldson P. M., Towrie M., Parker A. W., Baker M. J., Hunt N. T. (2019). Chem. Sci..

[cit28] Shaw D. J., Hill R. E., Simpson N., Husseini F. S., Robb K., Greetham G. M., Towrie M., Parker A. W., Robinson D., Hirst J. D., Hoskisson P. A., Hunt N. T. (2017). Chem. Sci..

[cit29] Farrell K. M., Ostrander J. S., Jones A. C., Yakami B. R., Dicke S. S., Middleton C. T., Hamm P., Zanni M. T. (2020). Opt. Express.

[cit30] Donaldson P. M., Greetham G. M., Shaw D. J., Parker A. W., Towrie M. (2018). J. Phys. Chem. A.

[cit31] Luther B. M., Tracy K. M., Gerrity M., Brown S., Krummel A. T. (2016). Opt. Express.

[cit32] Asplund M. C., Zanni M. T., Hochstrasser R. M. (2000). Proc. Natl. Acad. Sci. U. S. A..

[cit33] Donaldson P. M., Greetham G. M., Middleton C. T., Luther B. M., Zanni M. T., Hamm P., Krummel A. T. (2023). Accounts Chem. Res..

[cit34] Ren H., Zhang Q., Wang Z. J., Zhang G. Z., Liu H. Z., Guo W. Y., Mukamel S., Jiang J. (2022). Proc. Natl. Acad. Sci. U. S. A..

[cit35] Wu F., Huang Y., Yang G. K., Ye S., Mukamel S., Jiang J. (2024). Proc. Natl. Acad. Sci. U. S. A..

[cit36] Farmer A. L., Brown K., Hunt N. T. (2024). Vib. Spectrosc..

[cit37] Putnam F. W. , The Plasma Proteins, AP London, 2nd edn, 1975

[cit38] Hu S., Loo J. A., Wong D. T. (2006). Proteomics.

[cit39] Petricoin E. F., Belluco C., Araujo R. P., Liotta L. A. (2006). Nat. Rev. Cancer.

[cit40] Grosserueschkamp F., Bracht T., Diehl H. C., Kuepper C., Ahrens M., Kallenbach-Thieltges A., Mosig A., Eisenacher M., Marcus K., Behrens T., Bruning T., Theegarten D., Sitek B., Gerwert K. (2017). Sci. Rep..

[cit41] Sala A., Cameron J. M., Jenkins C. A., Barr H., Christie L., Conn J. J. A., Evans T. R. J., Harris D. A., Palmer D. S., Rinaldi C., Theakstone A. G., Baker M. J. (2022). Cancers.

[cit42] Cameron J. M., Rinaldi C., Rutherford S. H., Sala A., Theakstone A. G., Baker M. J. (2022). Appl. Spectrosc..

[cit43] Zigman M., Huber M., Kepesidis K., Voronina L., Fleischmann F., Fill E., Hermann J., Koch I., Kolben T., Schulz G. B., Jokisch F., Reinmuth N., Gesierich W., Behr J., Harbeck N., Reiser M., Stief C. G., Krausz F. (2022). Ann. Oncol..

[cit44] Voronina L., Leonardo C., Mueller-Reif J. B., Geyer P. E., Huber M., Trubetskov M., Kepesidis K. V., Behr J., Mann M., Krausz F., Zigman M. (2021). Angew. Chem., Int. Ed..

[cit45] Kepesidis K. V., Bozic-Iven M., Huber M., Abdel-Aziz N., Kullab S., Abdelwarith A., Al Diab A., Al Ghamdi M., Abu Hilal M., Bahadoor M. R. K., Sharma A., Dabouz F., Arafah M., Azzeer A. M., Krausz F., Alsaleh K., Zigman M., Nabholtz J. M. (2021). BMC Cancer.

[cit46] Huber M., Kepesidis K. V., Voronina L., Fleischmann F., Fill E., Hermann J., Koch I., Milger-Kneidinger K., Kolben T., Schulz G. B., Jokisch F., Behr J., Harbeck N., Reiser M., Stief C., Krausz F., Zigman M. (2021). Elife.

[cit47] Huber M., Kepesidis K. V., Voronina L., Bozic M., Trubetskov M., Harbeck N., Krausz F., Zigman M. (2021). Nat. Commun..

[cit48] Rutherford S. H., Nordon A., Hunt N. T., Baker M. J. (2021). Chemom. Intell. Lab. Syst..

[cit49] Board R. , Spalding K., Haworth E., Butler H. and Baker M. J., Presented in part at the 15th International Congress of the Society for Melanoma Research (SMR); Published in Pigment Cell and Melanoma research 2019 (3)1, p. 101, Manchester, 2018

[cit50] Weber J., Mandala M., Del Vecchio M., Gogas H. J., Arance A. M., Cowey C. L., Dalle S., Schenker M., Chiarion-Sileni V., Marquez-Rodas I., Grob J. J., Butler M. O., Middleton M. R., Maio M., Atkinson V., Queirolo P., Gonzalez R., Kudchadkar R. R., Smylie M., Meyer N., Mortier L., Atkins M. B., Long G. V., Bhatia S., Lebbe C., Rutkowski P., Yokota K., Yamazaki N., Kim T. M., de Pril V., Sabater J., Qureshi A., Larkin J., Ascierto P. A. (2017). N. Engl. J. Med..

[cit51] Luke J. J., Rutkowski P., Queirolo P., Del Vecchio M., Mackiewicz J., Chiarion-Sileni V., de la Cruz-Merino L., Khattak M. A., Schadendorf D., Long G. V., Ascierto P. A., Mandala M., De Galitiis F., Haydon A., Dummer R., Grob J. J., Robert C., Carlino M. S., Mohr P., Poklepovic A., Sondak V. K., Scolyer R. A., Kirkwood J. M., Chen K., Diede S. J., Ahsan S., Ibrahim N., Eggermont A. M. M., Investigators K. (2022). Lancet.

[cit52] Eggermont A. M. M., Blank C. U., Mandala M., Long G. V., Atkinson V. G., Dalle S., Haydon A. M., Meshcheryakov A., Khattak A., Carlino M. S., Sandhu S., Larkin J., Puig S., Ascierto P. A., Rutkowski P., Schadendorf D., Koornstra R., Hernandez-Aya L., Di Giacomo A. M., van den Eertwegh A. J. M., Grob J. J., Gutzmer R., Jamal R., Lorigan P. C., van Akkooi A. C. J., Krepler C., Ibrahim N., Marreaud S., Kicinski M., Suciu S., Robert C., Grp E. M. (2021). Lancet Oncol..

[cit53] Dummer R., Hauschild A., Santinami M., Atkinson V., Mandala M., Kirkwood J. M., Sileni V. C., Larkin J., Nyakas M., Dutriaux C., Haydon A., Robert C., Mortier L., Schachter J., Lesimple T., Plummer R., Dasgupta K., Gasal E., Tan M., Long G. V., Schadendorf D. (2020). N. Engl. J. Med..

[cit54] De Simoni E., Spagnolo F., Gandini S., Gaeta A., Rizzetto G., Molinelli E., Simonetti O., Offidani A., Queirolo P. (2024). Cancer Treat. Rev..

[cit55] Gupta R. B. A. , Corrie P., Hook J., Larkin J., Middleton M., Mowatt D., Nathan P., Plummer R., Shaw H., Waterston A. and Lorigan P., Follow Up of Cutaneous Melanoma in the UK 2022 – Postion Paper, Melanoma Focus, 2022, https://www.melanomafocus.org

[cit56] Eastwood J. B., Procacci B., Gurung S., Lynam J. M., Hunt N. T. (2024). ACS Phys. Chem. Au.

[cit57] Shim S. H., Zanni M. T. (2009). Phys. Chem. Chem. Phys..

[cit58] Deflores L. P., Nicodemus R. A., Tokmakoff A. (2007). Opt. Lett..

[cit59] Rutherford S. H., Greetham G. M., Parker A. W., Nordon A., Baker M. J., Hunt N. T. (2022). J. Chem. Phys..

[cit60] Hume S., Greetham G. M., Donaldson P. M., Towrie M., Parker A. W., Baker M. J., Hunt N. T. (2020). Anal. Chem..

[cit61] Ruiz-Perez D., Guan H. B., Madhivanan P., Mathee K., Narasimhan G. (2020). BMC Bioinf..

[cit62] Halder R. K., Uddin M. N., Uddin M. A., Aryal S., Khraisat A. (2024). J. Big Data.

[cit63] Hussain S. F. (2019). Expert Syst. Appl..

[cit64] Ghaddar B., Naoum-Sawaya J. (2018). Eur. J. Oper. Res..

[cit65] Wang X. Y. , IEEE International Joint Conference on Neural Networks (IJCNN), San Jose, CA, 2011

[cit66] Schwarz D. F., König I. R., Ziegler A. (2010). Bioinformatics.

[cit67] Yang F., Wang H. Z., Mi H., Lin C. D., Cai W. W. (2009). BMC Bioinf..

[cit68] Lee R. J., Gremel G., Marshall A., Myers K. A., Fisher N., Dunn J. A., Dhomen N., Corrie P. G., Middleton M. R., Lorigan P., Marais R. (2018). Ann. Oncol..

[cit69] Board R., Ellison G., Orr M., Kemsley K. R., McWalter G., Blockley L. Y., Dearden S. P., Morris C., Ranson M., Cantarini M. V., Dive C., Hughes A. (2009). Br. J. Cancer.

[cit70] Diem M. (2018). J. Biophot..

[cit71] Voronina L. , Huber M., Geyer P., Muller J., Leonardo C., Trubetskov M., Kepesidis K. V., Mann M., Krausz F. and Zigman M., IEEE Conference on Lasers and Electro-Optics Europe, Munich, Germany, 2019

[cit72] Dunkelberger E. B., Grechko M., Zanni M. T. (2015). J. Phys. Chem. B.

[cit73] Weeks W. B., Buchanan L. E. (2022). J. Phys. Chem. Lett..

[cit74] Beleites C., Neugebauer U., Bocklitz T., Krafft C., Popp J. (2013). Anal. Chim. Acta.

[cit75] Brereton R. G. (2006). Trac. Trends Anal. Chem..

[cit76] Farrell K. M., Fields C. R., Dicke S. S., Zanni M. T. (2023). J. Phys. Chem. Lett..

