# Supplementary material for: Machine-learning based classification of 2D-IR liquid biopsies enables stratification of melanoma relapse risk
Source: Chem Sci. 2025 Apr 7;16(19):8394–404. doi: 10.1039/d5sc01526j (PMC11983777; doi:10.1039/d5sc01526j)
Supplement: SC-016-D5SC01526J-s001 [file SC-016-D5SC01526J-s001.pdf]

ARTICLE

Machine-learning based classification of 2D-IR liquid biopsies enables stratification of melanoma relapse risk

Kelly Brown,[a] Amy Farmer,[a] Sabina Gurung,[a] Matthew J. Baker, [b] Ruth Board[c] and Neil T. Hunt*[a]

Supporting Information

Table S1: Breakdown of patient metadata relating to each of the three sample classes described in the main text

	Control	Developed Metastasis	Metastatic	Total
No of Male Patients	4	7	12	23
No of Female Patients	4	4	9	17
Age Range	43 - 83	46 - 72	21 - 88	21 - 88
Average Age	66.5	60.3	67.2	65.2
BRAF Negative	5	5	13	23
BRAF Postitive	3	6	8	17

a. Department of Chemistry and York Biomedical Research Institute, University of York, UK
b. School of Medicine and Dentistry, University of Central Lancashire, UK.
c. Department of Oncology, Lancashire Teaching Hospitals NHS Trust, Preston, UK.
** Corresponding author email: neil.hunt@york.ac.uk*
Supplementary Information available: [details of any supplementary information available should be included here]. See DOI: 10.1039/x0xx00000x

Please do not adjust margins

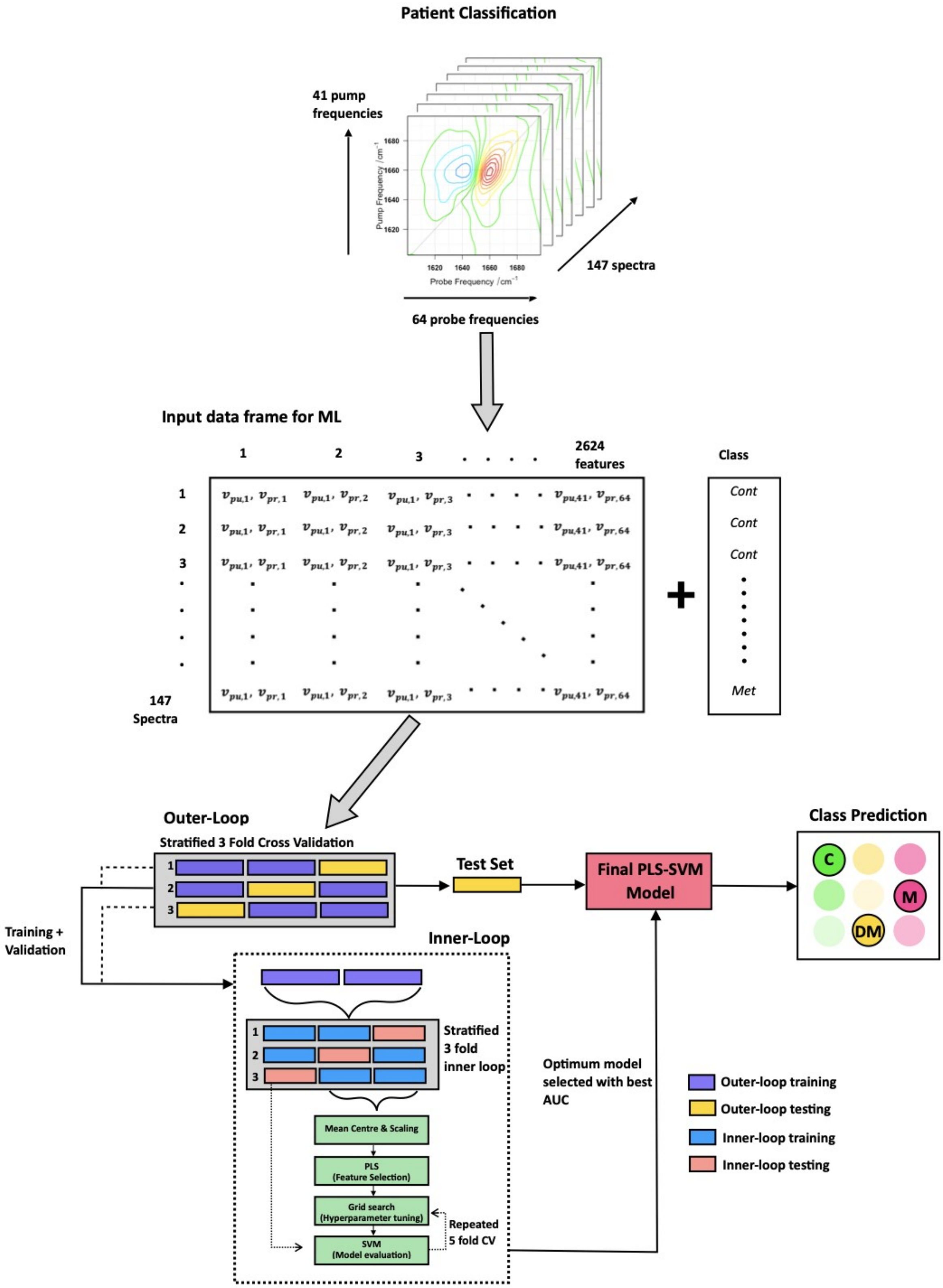

Figure S1: Schematic diagram showing construction of 2D-IR library and use of library within ML models described in the main text. Central panel shows the format of the data frame while the lower panel displays the model architecture including testing and training phases.

This journal is © The Royal Society of Chemistry 20xx

Please do not adjust margins

Please do not adjust margins

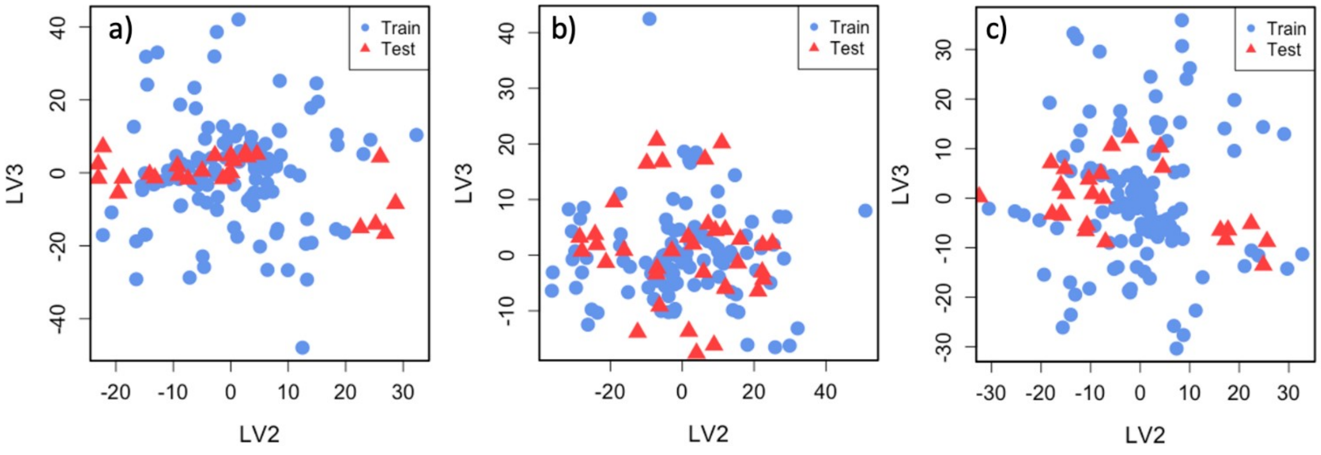

Figure S2: Scores plots for LV 2 vs LV 3 used to assess overlap between scores of the training and test sets within each outer fold, where a) is outer fold 1, b) is outer fold 2 and c) is outer fold 3, to check suitability of using independent PLS reductions on training and testing sets.

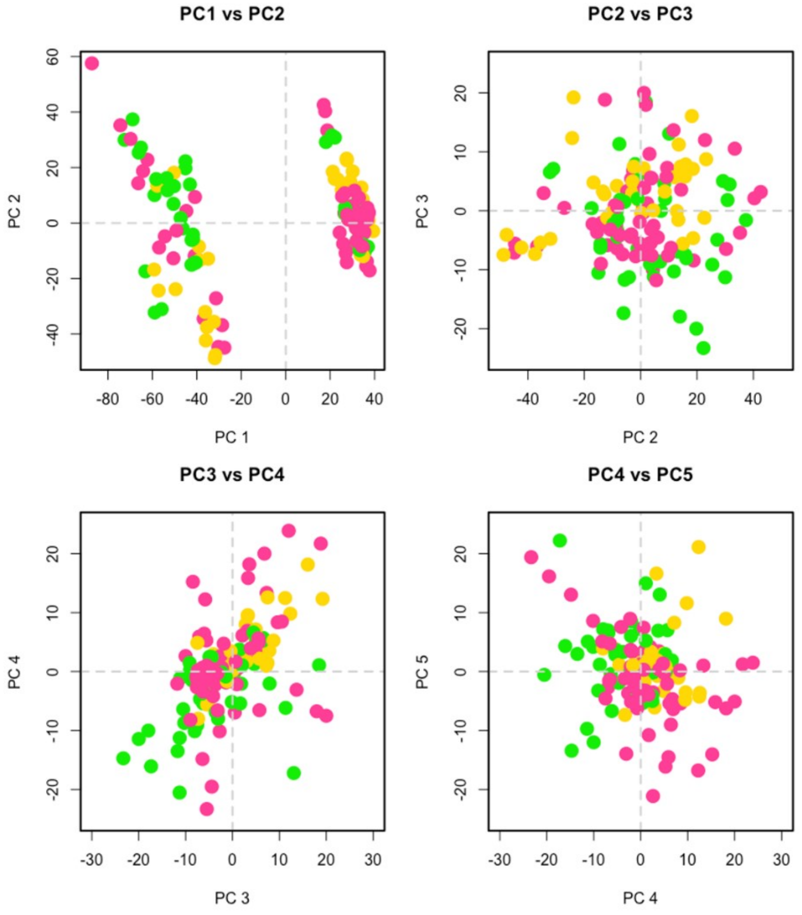

Figure S3: Scores plots for first four PCs from PCA analysis, where green = *control*, gold = *developed metastasis* and pink = *metastatic* groups. Plots highlight no distinct clustering between the three groups and significant overlap. Some subtle clustering can be observed in PC3 for example, indicating subtle spectral differences are likely apparent but require more advanced analysis techniques for extraction.

Please do not adjust margins

Please do not adjust margins

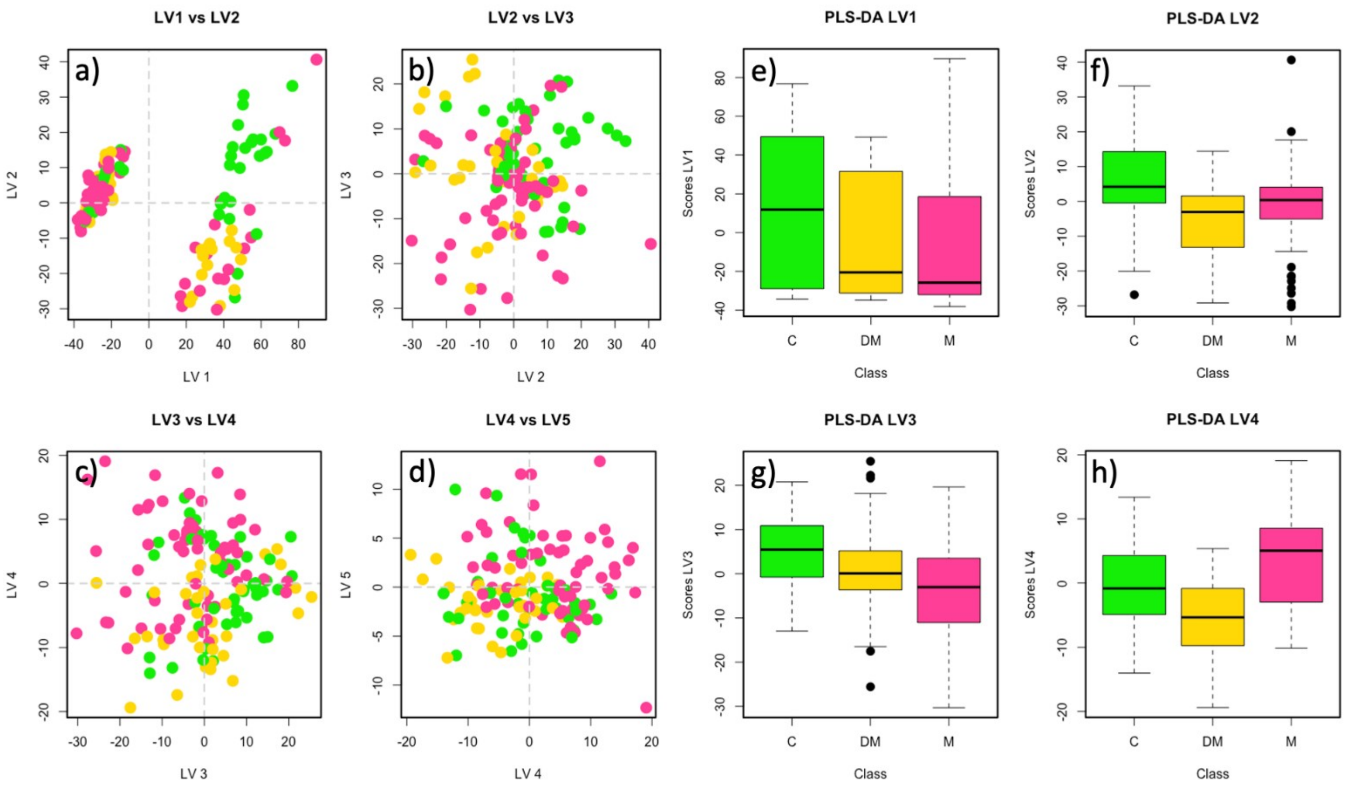

Figure S4: Scores plots for first four LVs from PLS-DA model, where green = *control*, gold = *developed metastasis* and pink = *metastatic* groups. a)-d) Plots compare scores for pairs of LVs while e)-h) show separations in LV scores between groups for a given LV. Distinct clustering and significant overlap is observed between the three groups. Some subtle clustering can be observed in LV3 for example, indicating spectral differences, but requiring more advanced analysis techniques for extraction.

This journal is © The Royal Society of Chemistry 20xx

Please do not adjust margins

Confusion Matrix

Predicted \ Actual	C	DM	M
C	8	0	2
DM	1	6	1
M	3	3	6

	Control	Developed Metastasis	Metastatic
Sensivity	0.667	0.667	0.667
Specificity	0.722	0.905	0.857
AUC	0.694	0.786	0.762
Kappa	0.492		
P-Value	2.854E-03		

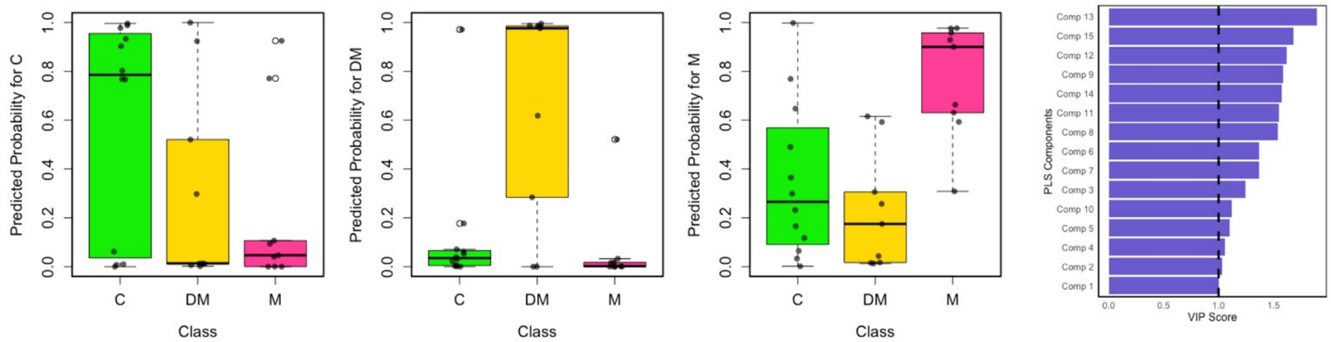

Figure S5: Summary of model performance for outer fold 1. Top row shows confusion matrix and performance statistics. Lower row shows prediction probability distribution plots for each class and latent variable importance plot.

Confusion Matrix

Predicted \ Actual	C	DM	M
C	11	0	3
DM	3	6	2
M	1	0	10

	Control	Developed Metastasis	Metastatic
Sensivity	0.733	1.000	0.667
Specificity	0.857	0.833	0.952
AUC	0.795	0.917	0.810
Kappa	0.621		
P-Value	5.126E-05		

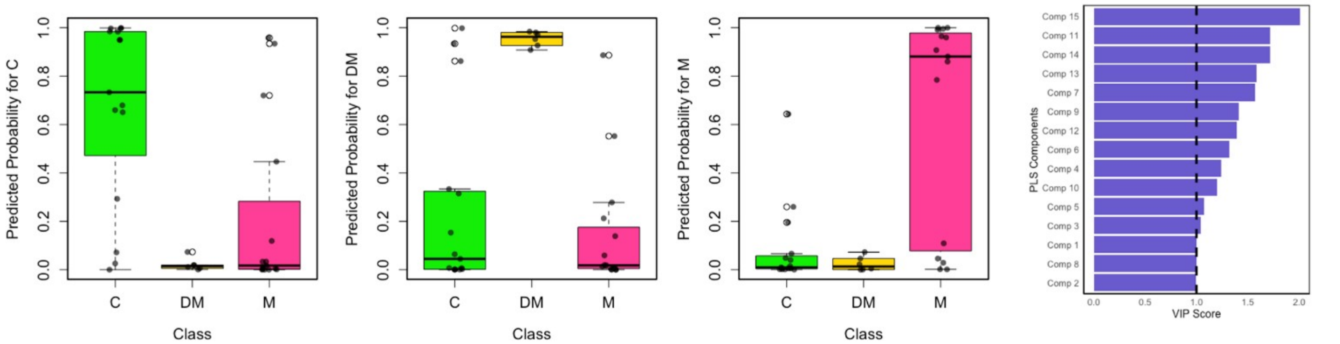

Figure S6: Summary of model performance for outer fold 2. Top row shows confusion matrix and performance statistics. Lower row shows prediction probability distribution plots for each class and latent variable importance plot.

Confusion Matrix

Predicted \ Actual	C	DM	M
C	4	2	2
DM	1	6	0
M	1	4	7

	Control	Developed Metastasis	Metastatic
Sensivity	0.667	0.500	0.778
Specificity	0.714	0.933	0.833
AUC	0.691	0.717	0.806
Kappa	0.455		
P-Value	4.101E-02		

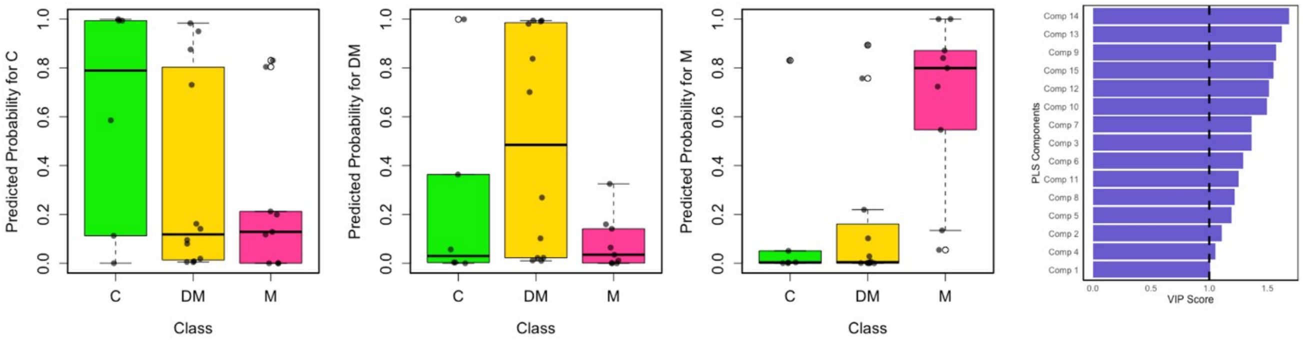

Figure S7: Summary of model performance for outer fold 3. Top row shows confusion matrix and performance statistics. Lower row shows prediction probability distribution plots for each class and latent variable importance plot.

This journal is © The Royal Society of Chemistry 20xx
